# Mechanism of swertiamarin and novel nitrogen-containing metabolites (*R*)-Gentiandiol and (*S*)-Gentiandiol in treating non-alcoholic fatty liver disease in rats: an untargeted metabolomics study based on UPLC-Q-TOF/MS

**DOI:** 10.3389/fphar.2025.1655026

**Published:** 2025-09-22

**Authors:** Yidan Sun, Fuyan Cui, Shuhan Tang, Pengyu Li, Yaqi Xu, Hao Li, Yige Wang, Xintong Li, Minyue Zhang, Rong Ma, Xianna Li, Hongying Xu, Ying Wang, Hailong Zhang, Zhigang Wang

**Affiliations:** ^1^ Department of Pharmaceutical Analysis, College of Pharmacy, Heilongjiang University of Chinese Medicine, Harbin, China; ^2^ Shangqiu Third People’s Hospital, Shangqiu, China; ^3^ Institute of Natural Medicine, University of Toyama, Toyama, Japan; ^4^ Heilongjiang Hospital, Beijing Children’s Hospital (Jiangnan Area, The Sixth Affiliated Hospital of Harbin Medical University), Harbin, China; ^5^ School of Pharmacy, Health Science Center, Xi’an Jiaotong University, Xi’an, Shaanxi, China

**Keywords:** Swertiamarin, (R)-Gentiandiol, (S)-Gentiandiol, non-alcoholic fatty liver disease, UPLC-Q-TOF/MS, Chinese medicine monomers

## Abstract

Swertiamarin, a predominant iridoid glycoside from hepatoprotective Swertia herbs, is biotransformed *in vivo* into nitrogen-containing metabolites (*R*)-gentiandiol and (*S*)-gentiandiol. These metabolites may be the real active hepatoprotective agents. A high-fat diet-fed rat model was treated for 12 weeks with swertiamarin, (*R*)-gentiandiol, (*S*)-gentiandiol and silybin, and the therapeutic effects on non-alcoholic fatty liver disease (NAFLD) were systematically evaluated through biochemical indices and histopathological observations. Swertiamarin and (*R*)-gentiandiol reversed high-fat diet-induced metabolic disturbances, reduced serum alanine aminotransferase, aspartate aminotransferase, total cholesterol, triglyceride, low-density lipoprotein cholesterol and malondialdehyde, while elevated high-density lipoprotein cholesterol, superoxide dismutase and glutathione peroxidase. However, (*S*)-gentiandiol exhibited no efficacy. The differential biomarkers in the serum of high-fat diet-fed rats were determined and identified by the metabolomics method combined with multivariate analysis. The results of enrichment analysis showed that NAFLD could be improved by swertiamarin and (*R*)-gentiandiol by regulating the levels of 21 biomarkers, such as stearic acid, palmitic acid and PC (36:3). According to the pathway enrichment results, swertiamarin and (*R*)-gentiandiol had potent combined effects in regulating taurine and hypotaurine metabolism, arachidonic acid metabolism, etc. This study is the first verification of the metabolite activity in the NAFLD model, and the dose-dependent effects of (*R*)-gentiandiol can be used to underscore its central role in swertiamarin’s bioactivity. These findings offer valuable insights to clarify the pharmaceutical material for hepatoprotective effect of *Swertia* herbs.

## 1 Introduction

As living standards have elevated globally, NAFLD has emerged as the predominant liver pathology, and this condition is intricately intertwined with systemic metabolic disorders. NAFLD refers to a complex spectrum of disorders characterized by hepatocellular damage, oxidative stress, and hepatic fibrosis, potentially culminating in the development of non-alcoholic steatohepatitis (NASH), liver cirrhosis, or eventually hepatocellular carcinoma (HCC) (Tsochatzis, 2022; Younossi et al., 2018). While NAFLD has a pervasive prevalence, no consensus on this condition has been reached, and the availability of specialized pharmaceutical interventions for NAFLD also remains elusive. In recent years, Chinese herbal extracts have been reported to exhibit excellent therapeutic effects in the context of treating NAFLD, indicating that Chinese herbal monomers could have the potential to exhibit efficacy on treating NAFLD with fewer negative effects (W. Zhao et al., 2016; H. Zhou et al., 2021; Zhu et al., 2025).

**FIGURE 1:**
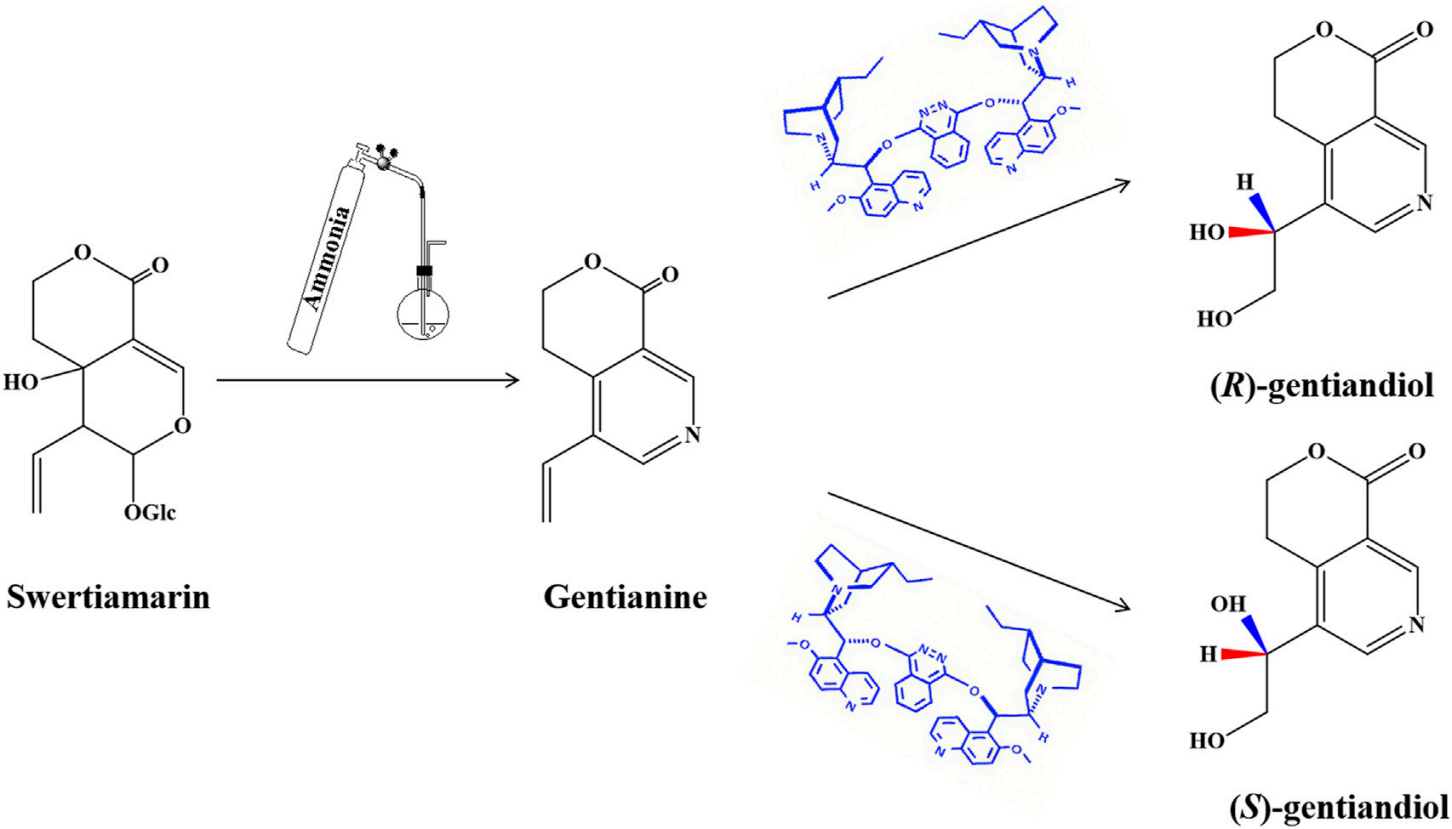
Synthetic routes of RG and SG.

*Swertia* herbs belong to the *Gentianaceae* family and are used extensively for treating digestive dysfunction hepatobiliary and metabolic disorders (Jauhari et al., 2017; Si et al., 2022). Swertiamarin (STM) is an iridoid glycoside that is the active component of *Swertia*. STM has various pharmacological effects, including anti-liver damage, resistance against hepatitis B virus, and hypolipidemic and hypoglycemic effects (Patel et et al., 2016; Xu et al., 2021). It has recently been suggested that STM could be used to treat NAFLD by reducing hepatic oxidative stress and lipid accumulation through the regulation of lipid metabolism and inflammatory responses.(Dai et al., 2018; Q. Yang et al., 2020; Yang et al., 2019). In a previous study, STM was hydrolyzed using *β*-glucosidase to an unstable genin *in vivo* and then was converted into *gentianine* under the action of the intestinal flora. Finally, the novel nitrogen-containing metabolites *(R)*-gentiandiol (RG) and *(S)*-gentiandiol (SG) were detected in rats after oral administation of STM using LC-MS and then synthesized from STM for the first time (Figure 1). This process demonstrated the transformation of STM into the novel nitrogen-containing metabolite gentiandiol *in vivo* (Z. G. Wang et al., 2012). The herbal monomers STM, RG, and SG are likely the main active ingredients of *Swertia* responsible for its therapeutic effect against NAFLD. However, the mechanism through which STM and its nitrogen-containing metabolites affect NAFLD has not been fully investigated to date, and an *in vivo* study of this mechanism is warranted.

Metabolomics as a technology provides an accurate identification and determination of metabolites using modern analytical techniques to explore the biomarkers and metabolic pathways associated with different diseases (Cheng et al., 2017). Metabolomics may be used to integrate the characteristics of Chinese medicine with medical research to investigate the development of diseases using a systematic and dynamic approach. This approach allows for elucidating the therapeutic mechanisms and the material basis of traditional Chinese medicine (TCM), thereby serving as a valuable tool for the modernization of TCM (Huang et al., 2017).

In this study, an NAFLD model was established in rats. Afterward, UPLC-Q-TOF/MS based on serum metabolomics was used to investigate the potential regulatory effects of STM and its nitrogen-containing metabolites on NAFLD and also reveal the potential metabolic pathways associated with NAFLD. In this way, the potential active metabolites of STM were identified.

## 2 Methods and materials

### 2.1 Drugs and reagents

The normal diet for rats was purchased from Changsheng Biotechnology Company (Liaoning, China). The high-fat diet for rats was self-produced (ingredients: 77.5% normal diet, 10% yolk powder, 10% lard, 2% cholesterol, 0.5% bile salt). Silybin was purchased from Tianshili Shengte Pharmaceutical Company (Tianjin, China). Acetonitrile (UPLC grade) and formic acid (UPLC grade) were purchased from Merck Group (Darmstadt, Germany). Swertiamarin (STM, chemical formula C_16_H_22_O_10_, molecular weight 374.43, Cat. CAS 17388–39-5) at 98.04% purity was obtained from the Master of Bioactive Molecules. RG and SG were synthesized as previously described (Z. G. Wang et al., 2012). The assay kits for total cholesterol (TC), triglyceride (TG), alanine aminotransferase (ALT), aspartate aminotransferase (AST), low-density lipoprotein cholesterol (LDL-C), high-density lipoprotein cholesterol (HDL-C), malondialdehyde (MDA), superoxide dismutase (SOD), and glutathione peroxidase (GSH-PX) were acquired from Nanjing Jiancheng Biological Engineering Institute (Nanjing, China). Milli-Q ultrapure water system (Billerica, USA) was used for purifying distilled water. Ammonia, hydrochloric acid, chloroform, petroleum benzene, sodium sulfite, ethyl acetate, and asymmetric dihydroxylation (AD) mixtures were acquired from Sigma Chemical (St. Louis, MO, USA).

### 2.2 Animal experiments and sample collection

Adult male Sprague-Dawley rats weighing 200 ± 20 g were acquired from the Animal Breeding Center at the Heilongjiang University of Traditional Chinese Medicine (Heilongjiang, China) [License No. SCXK (Hei) 2018–003]. The animal study was approved by the Ethics Committee of the Heilongjiang University of Chinese Medicine [Resolution No. 2023012702] and followed the Declaration of Helsinki. All acquired rats were maintained under standard laboratory conditions (23 °C–25 °C, 12-h/12-h light/dark cycle, 55% ± 2% humidity) for 1 week for acclimation, with unrestricted access to water and food. All animals were randomly divided into eight groups: the control, model, positive, STM, RG high-dose (RGH), RG medium-dose (RGM), RG low-dose (RGL), and SG groups (n = 10 each).

**FIGURE 2:**
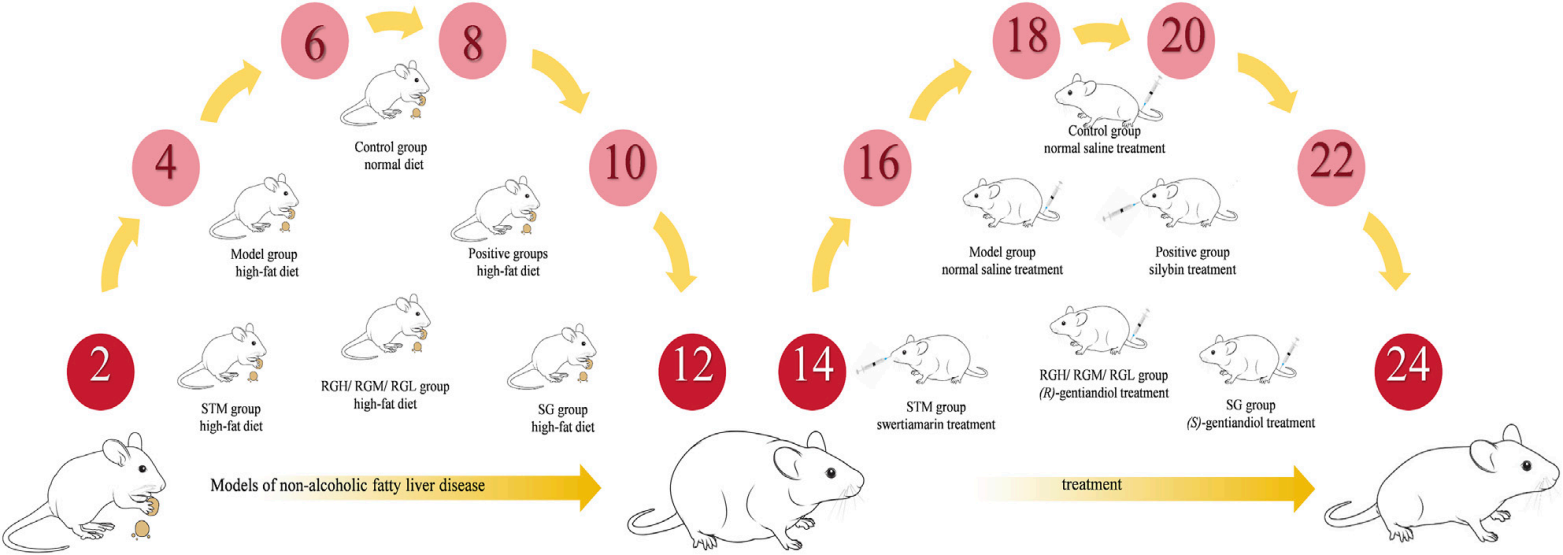
Modeling and treatment procedures.

A normal diet was given to the control group, while an HFD was given to the other seven groups for 12 weeks. After model establishment, the STM group was administered STM orally at a dosage of 25 mg/kg, while the RGH group was intravenously injected with 6 mg/kg RG (in saline suspension). The RGM group was intravenously injected with 3 mg/kg RG (in saline suspension), the RGL group was intravenously injected with 1.5 mg/kg RG (in saline suspension), and the SG group was intravenously injected with SG (6 mg/kg, suspended in saline). The positive control group was administered silybin (37.8 mg/kg) by gavage. 0.9% saline (10 mL/kg) was administered intravenously to both the model and control groups. All animals received treatment once daily for 12 weeks (Figure 2), after which the rats were subjected to 24 h of fasting before blood was collected from the hepatic portal vein. The blood sample was centrifuged (3500 rpm, 4 °C, 15 min) to separate the serum, which was preserved at −80 °C until biochemical and metabolomic analyses were conducted. The liver samples retrieved from the rats were also stored immediately at −80 °C for backup.

### 2.3 Histopathological assessments

A portion of the left lobe liver tissue was excised and immersed in 10% buffered formalin solution for 48 h, followed by dehydration and paraffin wax embedding. After the hematoxylin-eosin (HE) staining of paraffin-embedded samples, the changes in each group of hepatocytes were observed under a light microscope. Oil Red O solution was added to stain the frozen liver tissues, followed by observation under a light microscope. The grading was defined by a pathologist blinded to the treatment according to the NAFLD activity score (NAS). NAS is the sum of the separate scores for steatosis(0–3), hepatocellular ballooning(0–2), and lobular inflammation(0–3). Samples with scores more than 5 were associated with a diagnosis of NASH, and score less than 3 were diagnosed as not NASH (Kleiner et al., 2005).

### 2.4 Determination of biochemical indices

TC, TG, ALT, AST, LDL-C, and HDL-C were determined using the corresponding kits and 100 µL serum samples, by strictly following kit instructions. The liver sample was proportionally added to saline at a 9-fold volume and homogenized on an ice water bath. After 10 min of centrifugation (13,000 rpm, 4 °C), the supernatants were collected, and the MDA, SOD, and GSH-PX levels were measured using the corresponding kits.

### 2.5 Preparation of serum samples

The frozen serum samples were thawed at 4 °C. A total of 100 µL of serum was diluted with analytical-grade methanol (100%, 4 °C) to a total volume of 400 μL, followed by 1 min of vortex mixing. The mixture was subsequently centrifuged (13,000 rpm, 4 °C, 20 min), and the obtained supernatants were filtered through a 0.22 µm microfiltration membrane and added to an injection vial for a UPLC-MS analysis. All samples were mixed in equal volumes (10 μL) and used as quality control samples.

### 2.6 UPLC-Q-TOF/MS analysis

Separation was completed using a Thermo ScientificTM ultrahigh-performance liquid chromatography (UPLC) system (Thermo, San Jose, CA, USA). Chromatography was performed on a Waters ACQUITY UPLC CSH C_18_ chromatographic column (1.7 μm, 2.1 mm × 100 mm; Waters, Milford, USA) at a column temperature of 35 °C, sample manager temperature of 4 °C, injection volume of 3 μL, and flow rate of 0.3 mL/min. The mobile phase contained solvent A (0.1% formic acid-water) and solvent B (0.1% formic acid-acetonitrile), and elution was performed under the following conditions: 0–4 min, 5%–40% B; 4–7 min, 40%–55% B; 7–11 min, 55%–65% B; 11–15 min, 65%–100% B; 15–15.1 min, 100%–5% B; 15.1–16 min, 5%–5% B. The UPLC system was connected to a mass spectrometer.

Mass spectrometry was conducted using a Q Exactive Orbitrap mass spectrometer (Thermo, San Jose, CA, USA). The electrospray ionization (ESI) source conditions were as follows: the scan mass range was 100–1,500 Da; the sheath, aux, and sweep gas flow rates were 50, 15, and 1 Arb, respectively. The capillary voltages were 500 V and 3,000 V in positive-ion and negative-ion modes, respectively, whereas the capillary temperature was 350 °C. In order to ensure detection system stability and the reproducibility of detection, QC was also added in sequence at 10 sample intervals.

### 2.7 Multivariate data analysis

Compound Discoverer 3.1 software (Thermo Fisher, USA) was used to extract and filter the LC-MS data and match and normalize the peaks, thus obtaining the compound molecular weights, peak areas, and retention times for every sample. The normalized datasets of ESI^−^ and ESI^+^ modes were combined and input into SIMCA 13.0 software (MKS Umetrics, Malmö, Sweden) to conduct various multivariate data analyses, namely, principal component analysis (PCA), partial least squares discriminant analysis (PLS-DA), and orthogonal partial least squares discriminant analysis (OPLS-DA). Consequently, variables with VIP >1 were identified as the significant variables of potential endogenous metabolites. The results were presented as the means ± standard deviations and were analyzed using SPSS statistical software 25.0 (IBM SPSS, Chicago, USA). The intra-group differences were analyzed using one-way ANOVA and Tukey’s test. *P* < 0.05 indicated statistical significance. Finally, the VIP and t-test results (*P* < 0.05) were combined for biomarker identification.

### 2.8 Biomarker identification and pathway analysis

The molecular ions and predicted molecular formulas were compared with those in the ChemSpider (https://www.chemspider.com/) and *mz*Cloud (https://www.mzcloud.org/) databases for initial metabolite identification. In the next step, Thermos' Xcalibur (version 4.3) software was employed to compare the molecular weights, MS/MS ions, and retention times of the endogenous compounds with the standards and literature reports. Identification of metabolite structures and clarification of metabolite biological significance in the HMDB (https://hmdb.ca/) and KEGG (https://www.kegg.jp/kegg/kegg1.html) databases were performed to map comprehensive metabolic networks. Finally, the identified biomarkers were imported into MetaboAnalyst 5.0 (https://www.metaboanalyst.ca/) to reveal the enriched pathways.

## 3 Results

### 3.1 Effects on body weight and liver indices

**FIGURE 3:**
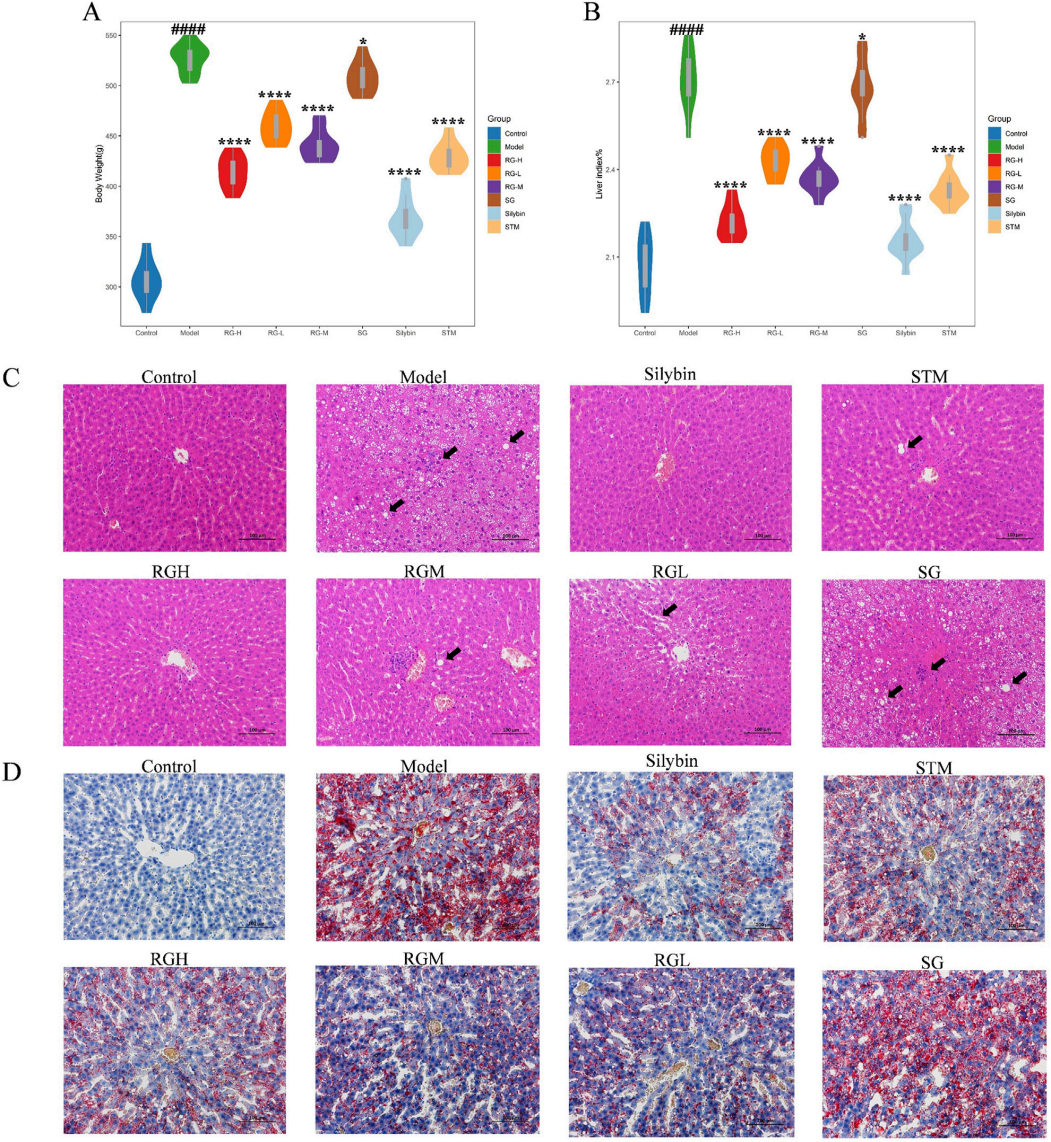
Body weight, hepatic index, and histopathological results of NAFLD model rats after administration of treatment in each group. **(A)** Body weight changes in NAFLD model rats; **(B)** Hepatic index changes in NAFLD model rats; **(C)** Liver histomorphologic changes were assessed by HE staining (200X); **(D)** Liver histomorphologic changes were assessed by Oil Red O staining (200X). ####p < 0.001 compared with normal control rat group. *p < 0.05, ****p < 0.01 compared with rat model of nonalcoholic fatty liver group.

The three herbal monomer extracts were administered to rats continuously for 12 weeks, and the rats remained healthy, active, and did not die during the study. As shown in Figure 3A, the body weight of the NAFLD model group apparently increased (*P* < 0.01) in comparison with that of the control group, whereas the body weight gradually decreased after treatment with STM, RGH, RGM, and RGL. The RGH group exhibited the most significant improvement, while no significant decrease was noted in the SG group.

Rat liver indices of the different groups were calculated. As shown in Figure 3B, the liver indices of the NAFLD model group were markedly elevated (*P* < 0.01) relative to those of the control group, indicating that the HFD caused liver enlargement in rats after 12 weeks. After the administration of STM, RGH, RGM, or RGL, the liver indices of the rats dramatically declined (*P* < 0.01). The RGH group presented the most significant improvements, while no significant decrease was observed in the SG group.

### 3.2 Histopathological observations

Histopathological results helped to visualize the therapeutic effects of the three herbal monomers on HFD-induced NAFLD. As shown in Figure 3C, in the control group of rats, the hepatic lobules were normal, with clear hepatic sinusoids and normal nuclei. There was no hepatic steatosis or inflammatory cell infiltration. The rats in the model group presented damaged hepatic lobules, with dilated hepatic sinusoids, hepatic steatosis, and missing nuclei, which are typical of liver injury. Moreover, the inflammatory cell infiltrate was obvious. These findings coincided with the histopathological observations of NAFLD, suggesting that the HFD-induced NAFLD model was successfully established in this study. Inflammatory cell infiltration and lipid droplet formation in hepatocytes improved after treatment with STM, RGH, RGM, or RGL. Different treatment groups showed different levels of improvement, and each presented a reduction in cell necrosis rates and alleviation of hepatic steatosis. Notably, the RGH group presented the most significant ameliorative effects. No improvement was observed in the SG group. The NAS score results are presented in Table 1. As shown in Figure 3D, the percentage Oil Red O-stained area in the model group was dramatically greater than that in the control group, whereas the corresponding percentages in the STM, RGH, RGM, and RGL groups were markedly lower than those in the model group. The above findings indicated that the NAFLD model was successfully established and that STM, RGH, RGM, and RGL were effective against NAFLD. The RGH group showed the most significant improvement, while no significant improvement was observed in the SG group.

**TABLE 1:** NAS scores.

Histological feature Group	Steatosis	Lobular inflammation	Hepatocellular ballooning	NAS
Control	0	0	0	0
Model	2	1	1	4
Silybin	0	0	0	0
STM	1	0	1	2
RGH	0	0	0	0
RGM	1	1	0	2
RGL	1	1	0	2
SG	2	1	1	4

### 3.3 Effects on biochemical indices

**FIGURE 4:**
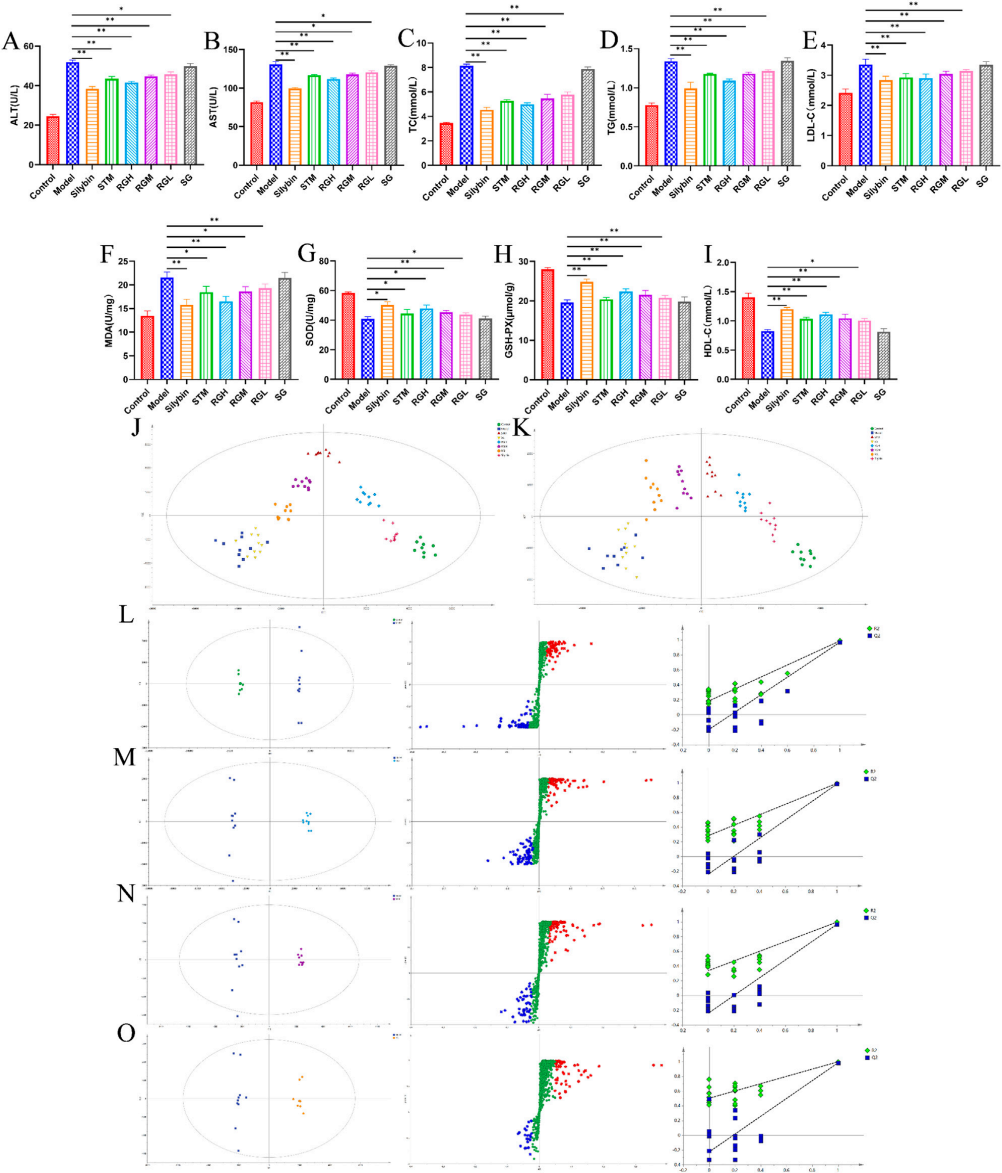
Biochemical indexes result of NAFLD model rats after administration of treatment in each group. **(A–I)** Biochemical parameters of serum ALT, AST, TC, TG, LDL-C, MDA, SOD, GSH-PX and HDL-C in the control, model, STM, RGH, RGM, RGL, SG and Silybin groups. *p < 0.05, **p < 0.01 represent data vs. the model group. **(J)** PLS-DA 2D score plots for eight groups of sera in ESI^+^. **(K)** PLS-DA 2D score plots for eight groups of sera in ESI^−^. **(L–O)** The OPLS-DA score plot, S-plot and corresponding coefficient of loading plots between the control and model groups; the RGH and model groups; the RGM and model groups; the RGL and model groups.

As shown in Figures 4A–I, the levels in the model group were significantly different from those in the control group. Specifically, the levels of MDA, AST, ALT, TC, TG, and LDL-C dramatically increased (*P < 0.01*), whereas the levels of SOD, GSH-PX, and HDL-C in the model group clearly decreased (*P < 0.01*). The index levels showed varying degrees of improvement after the administration of STM, RGH, RGM, and RGL. Compared to the other groups, the RG group exhibited greater improvement, whereas the SG group presented no improvement.

### 3.4 Metabolomics analysis and identification of biomarkers

As shown in Figures 4J,K, PLS-DA was used to compare the metabolic profiles of the six groups of rats. The samples from the control group and the model groups were aggregated on both sides. After administration, the STM, RGH, RGM, and RGL groups were separated from the model group and were approaching the control group. Notably, the RGH group was closest to the control group but far from the model group. The SG group was closer to the model group but far from the control group.

As shown in Figures 4L–O, the control and model groups, as well as the RG and model groups, were distinctly separated, further illustrating the significant discrepancies between different groups. The R^2^ and Q^2^ values of the OPLS-DA model were 0.996 and 0.991, respectively, for the control group, relative to the model group. For the OPLS-DA model, the R^2^ and Q^2^ values for RGH relative to the model groups were 0.994 and 0.987, respectively, while those for RGM relative to the model group were 0.976 and 0.944, respectively, and those for RGL relative to the model groups were 0.934 and 0.916, respectively. The corresponding S-PLOT plot is shown in Figures 4L–O. A total of 200 repeated permutation tests were performed for each data model, and the Q^2^ and R^2^ values obtained after permutation were apparently decreased relative to their initial values, indicating that the models were stable. Because the RGH group was the most representative, only the RGH group (RG) was selected for subsequent metabolomic analysis.

### 3.5 Metabolic pathway analysis

**FIGURE 5:**
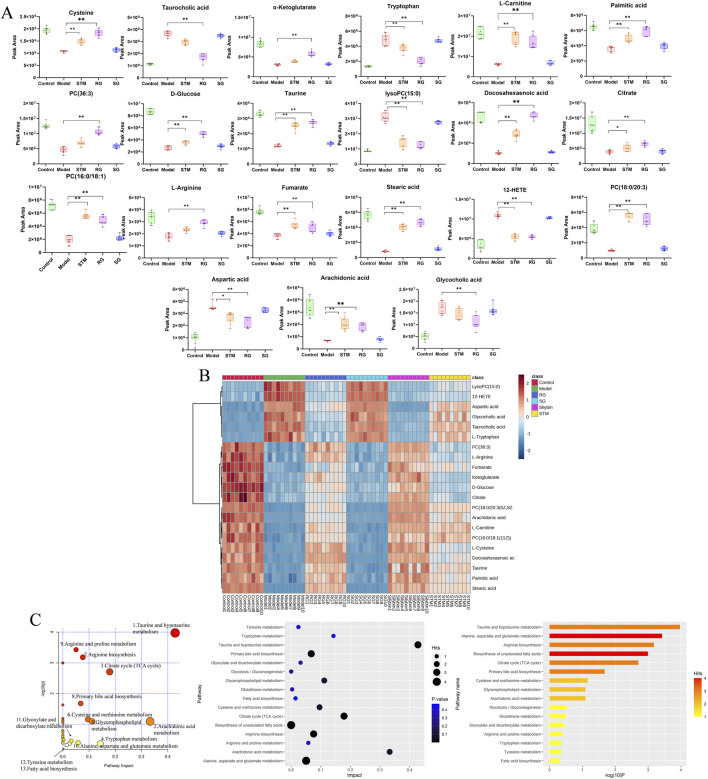
The serum metabolites in NAFLD model rats and metabolite pathway enrichment analysis based on biomarkers in the serum. **(A)** Variations in serum biomarker levels. *P* < 0.05, *P < 0.01. **(B)** Hierarchical clustering heat map of the 21 differential metabolites after treatment among six groups, with the degree of change marked by colour, including upregulation (red) and downregulation (blue). **(C)** Metabolite pathway enrichment analysis based on biomarkers in the serum.

A total of 21 differentially abundant metabolites were detected. Biomarker information and levels are presented in Table 2. Figures 5A,B shows the changes in the levels of 21 differential metabolites in each group along with a heatmap. Compared to those in the control group, the levels of lipid metabolites such as 12-HETE and lysoPC (15:0) were significantly increased, and the levels of docosahexaenoic acid, stearic acid, palmitic acid, PC (36:3), PC (16:0/18:1(11Z)), PC (18:0/20:3(5Z,8Z,11Z)), and arachidonic acid metabolites were significantly decreased. Moreover, arachidonic acid metabolites were significantly downregulated; the levels of amino acid metabolites such as L-tryptophan, aspartic acid were significantly upregulated, as well as L-arginine, L-cystine, and taurine were significantly decreased, the levels of bile acid metabolites taurocholic acid, and glycocholic acid were significantly increased, and the levels of D-glucose and L-carnitine were significantly decreased compared to the control group. Seventeen differential metabolites were regulated after STM. Twenty-one differential metabolites were regulated after RG. The therapeutic effect of SG on abnormal metabolites converged in the model.

**TABLE 2:** List and change trend of differential metabolites. “↑or↓” indicates increase or decrease, “–” indicates no significance, *p < 0.05, **p < 0.01 compared with the model group.

RT (min)	Ionisation mode	Metabolites	M/Z	Molecular formula	HMDB	Trend			Pathway
						M vs C	STM vs M	RG vs M	
2.47	ESI-	L-Tryptophan	204.2148	C11H12N2O2	HMDB0000929	Up	Down	Down	4
2.81	ESI-	L-Carnitine	162.2659	C7H16NO3	HMDB0000062	Down	Up	Up	–
3.75	ESI-	L-Arginine	174.2976	C6H14N4O2	HMDB0000517	Down	Flat	Up	7,10
4.05	ESI-	L-Cysteine	121.3487	C3H7NO2S	HMDB0000574	Down	Up	Up	1,6
4.84	ESI-	Aspartic acid	133.4795	C4H7NO4	HMDB0006483	Up	Down	Down	9
5.07	ESI-	Taurine	125.4927	C2H7NO3S	HMDB0000251	Down	Up	Up	1,8
7.38	ESI-	Arachidonic acid	304.5169	C20H32O2	HMDB0001043	Down	Up	Up	2
8.03	ESI-	Taurocholic acid	515.7843	C26H45NO7S	HMDB0000036	Up	Flat	Down	8,12
8.25	ESI+	12-HETE	320.4876	C20H32O3	HMDB0006111	Up	Down	Down	—
8.47	ESI-	Glycocholic acid	465.6359	C26H43NO6	HMDB0000138	Up	Flat	Down	8
9.08	ESI-	Fumarate	116.1845	C4H4O	HMDB0000134	Down	Up	Up	7,3,9
9.58	ESI-	Citrate	192.1876	C6H8O7	HMDB0000094	Down	Up	Up	3,9,11
10.75	ESI+	Docosahexaenoic acid	328.4769	C22H32O2	HMDB0002183	Down	Up	Up	—
11.08	ESI-	D-Glucose	180.1498	C6H12O6	HMDB0000122	Down	Up	Up	—
12.74	ESI-	α-Ketoglutarate	146.1084	C5H6O5	HMDB0000208	Down	Flat	Up	3,9,7
13.08	ESI+	Stearic acid	284.2978	C18H36O2	HMDB0000827	Down	Up	Up	—
13.71	ESI+	Palmitic acid	256.4179	C16H32O2	HMDB0000220	Down	Up	Up	13
13.85	ESI+	LysoPC (15:0)	481.6894	C23H48NO7P	HMDB0010381	Up	Down	Down	5
13.98	ESI+	PC (36:3)	784.2948	C44H82NO8P	HMDB0007921	Down	Flat	Up	2,5
14.03	ESI+	PC [16:0/18:1(11Z)]	760.1498	C42H82NO8P	HMDB0007971	Down	Up	Up	2,5
14.25	ESI+	PC [18:0/20:3(5Z,8Z,11Z)]	812.2487	C46H86NO8P	HMDB0008046	Down	Up	Up	2,5

**FIGURE 6:**
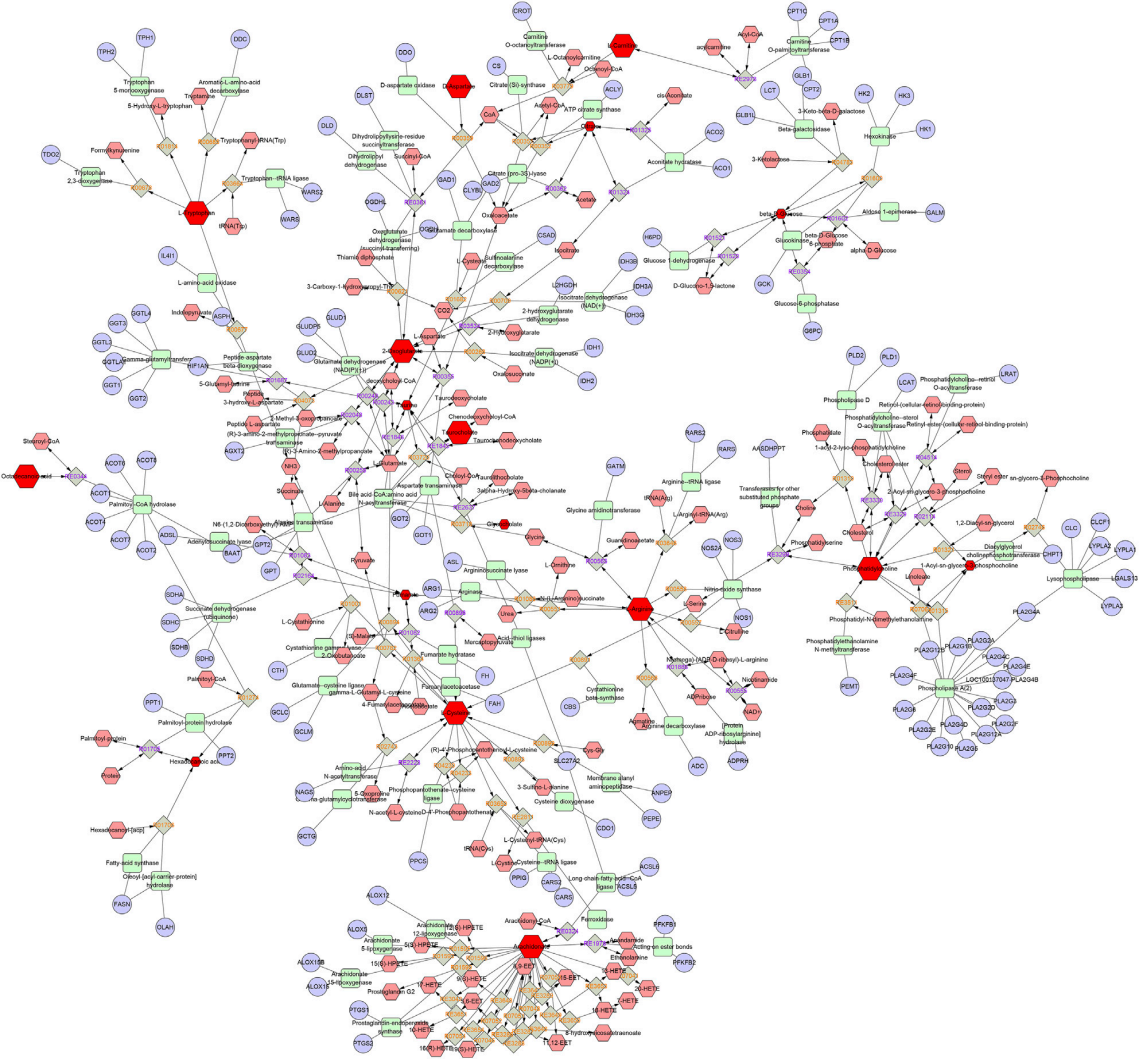
The metabolic network diagram. Blue represents the relevant target, pink represents other metabolites in this pathway, and red represents the differential metabolites identified in this experiment.

The metabolites involved in each metabolic pathway along with their details are listed in Table 3. Figure 5C shows the 14 metabolic pathways associated with the therapeutic mechanism of RG in NAFLD. According to the criterion of an impact >0.1, arachidonic acid metabolism, tryptophan metabolism, the TCA cycle, taurine and hypotaurine metabolism, and glycerophospholipid metabolism were identified as the significantly relevant metabolic pathways for this mechanism, and these pathways are important for NAFLD. A metabolic network diagram of 21 differentially abundant metabolites obtained from the RG group and model group is shown in Figure 6.

**TABLE 3:** Result from pathway analysis between RG and model groups.

Pathways	Total	Expected	Hits	Raw P	Holm adjust	FDR	Impact
Taurine and hypotaurine metabolism	8	0.10603	3	0.000107	0.008992	0.008992	0.42857
Arachidonic acid metabolism	36	0.47714	2	0.080362	1	0.046253	0.33292
Citrate cycle (TCA cycle)	20	0.26508	3	0.001968	0.15743	0.033061	0.17875
Tryptophan metabolism	41	0.54341	1	0.42562	1	1	0.14305
Glycerophospholipid metabolism	36	0.47714	2	0.080362	1	0.046253	0.11182
Cysteine and methionine metabolism	33	0.43738	2	0.068956	1	0.046253	0.09592
Arginine biosynthesis	14	0.18555	3	0.000661	0.05422	0.018514	0.07614
Primary bile acid biosynthesis	46	0.60968	3	0.021017	1	0.28254	0.06809
Arginine and proline metabolism	38	0.50364	1	0.40153	1	0.033061	0.05786
Alanine, aspartate and glutamate metabolism	28	0.37111	4	0.000375	0.031157	0.015766	0.05048
Glyoxylate and dicarboxylate metabolism	32	0.42412	1	0.35042	1	0.046253	0.03175
Tyrosine metabolism	42	0.55666	1	0.43345	1	0.046253	0.02463
Fatty acid biosynthesis	47	0.62293	1	0.47108	1	1	0.01472
Glutathione metabolism	28	0.37111	1	0.31408	1	1	0.00343
Glycolysis/Gluconeogenesis	26	0.3446	1	0.29519	1	1	0.00021
Biosynthesis of unsaturated fatty acids	36	0.47714	4	0.001008	0.081671	0.021174	0
Aminoacyl-tRNA biosynthesis	48	0.63618	3	0.023545	1	0.028254	0
Linoleic acid metabolism	5	0.066269	1	0.064619	1	0.046253	0
D-Glutamine and D-glutamate metabolism	6	0.079523	1	0.077058	1	1	0
alpha-Linolenic acid metabolism	13	0.1723	1	0.15983	1	0.95897	0

## 4 Discussion

NAFLD is a metabolic disease that disrupts body fat metabolism, increases the production of active oxygen, causes oxidative stress, and elicits an inflammatory response (Paradies et al., 2014). In recent years, research on TCM has shifted from clinical efficacy assessment to the study of the active components of TCM, and significant progress has been achieved in regard to several metabolic diseases (X. Liu et al., 2019; Xiao et al., 2013). Several experimental studies have demonstrated the hepatoprotective and anti-inflammatory therapeutic effects of STM (Mihailović et al., 2013; Wu et al., 2017). However, the precise mechanism remains unclear to date. A previous study by our research group revealed that STM is converted to nitrogenous metabolites *in vivo*, which maybe the real active hepatoprotective material.

In this study, the effects of three herbal monomers on NAFLD rat models were analyzed by synthesizing novel nitrogen-containing metabolites RG and SG from STM. The histological results revealed that the rats fed an HFD for 12 weeks presented numerous fat vacuoles and inflammatory infiltrates, with typical hepatic steatosis. Moreover, it was observed that the liver weight, body weight, and liver indices of the model rats had increased linearly, while their serum biochemical and antioxidant indices were at significantly abnormal levels. The above findings suggested that the NAFLD rat model was successfully established in this study. After RG treatment for 12 weeks, the liver index significantly decreased, accompanied by a substantial decrease in hepatic steatosis and a notable improvement in NAFLD symptoms. The abnormal levels of metabolites were effectively reversed after 12 weeks of treatment. The differentially abundant metabolite levels of the SG group were closer to those of the model group, suggesting that SG was largely ineffective against NAFLD.

**FIGURE 7:**
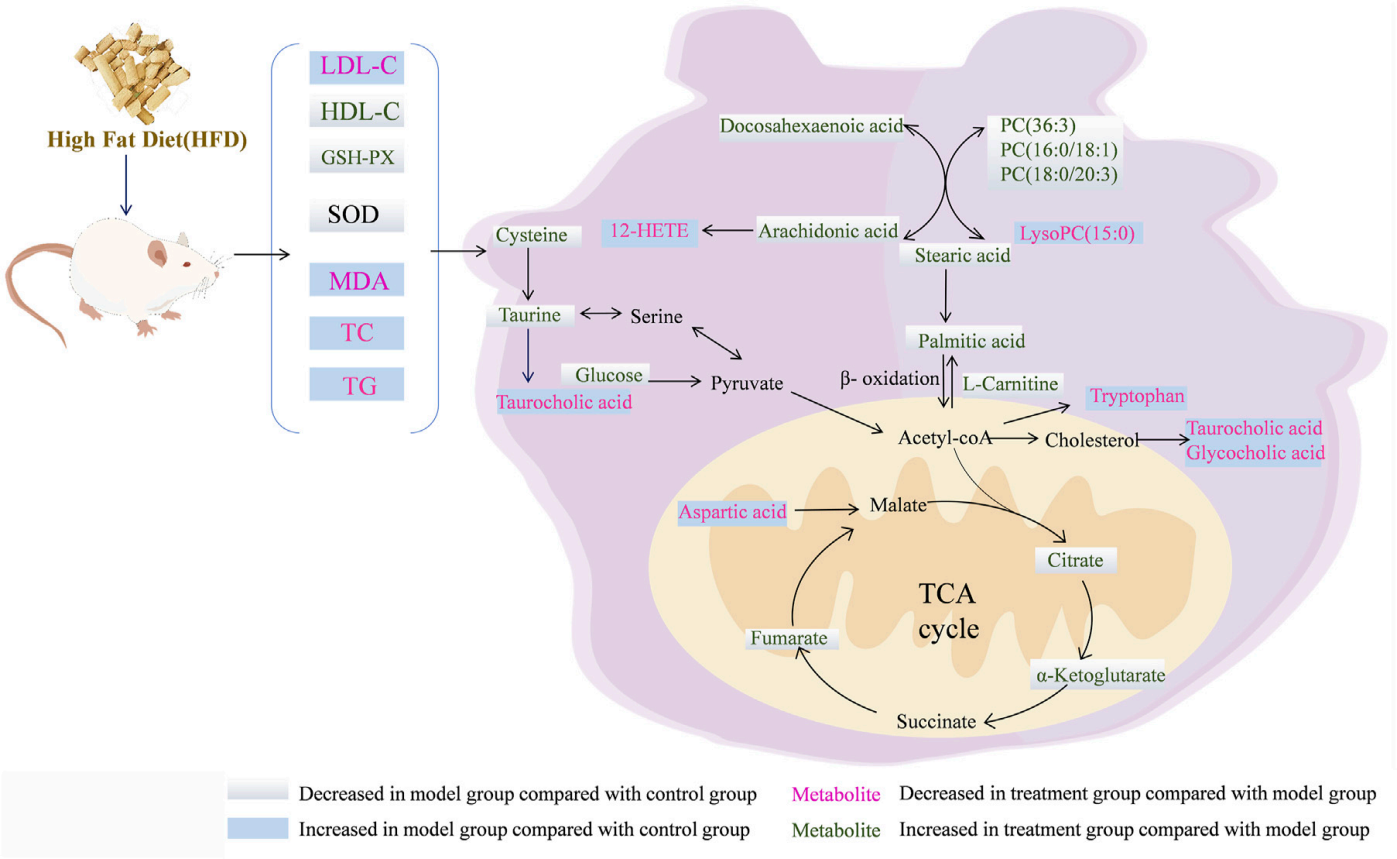
Overview of the metabolites and the major metabolic pathways in the NAFLD model after treatment with the RG.

Taurine has various physiological effects, including the inhibition of fatty acid-mediated lipid accumulation, reduction of reactive oxygen species generation, and improvement of fatty acid-induced damage to the inner mitochondrial membrane (Miyata et al., 2020). Taurine reportedly possesses therapeutic potential for liver fat accumulation, cardiovascular disease, and diabetes (Miyazaki and Matsuzaki, 2014; Wen et al., 2019). As rate-limiting enzymes, cysteine dioxygenase (CDO) regulates taurine synthesis, while glutamate-cysteine ligase (GCL) can regulate the metabolism of cysteine to glutathione (Ahn et al., 2015). In this study, the taurine and hypotaurine metabolic pathways of the model group were disrupted compared to those of the control group. The cysteine and taurine levels were decreased, and the taurocholic acid levels were elevated Figure 7. Accordingly, it was inferred that the inhibition of CDO and GCL activities suppressed taurine and glutathione synthesis, which aggravated hepatic oxidative damage and affected hepatic lipid metabolism. After treatment in the three groups, CDO and GCL activities may have increased, and the levels of taurine and cysteine were elevated. These results indicate that STM and RG improve antioxidant capacity by regulating the taurine and hypotaurine metabolic pathways, thereby effectively preventing the biofilm damage caused by lipid peroxidation and ultimately treating chronic liver injury. Taurocholic acid serves as a common primary bile acid derived from the combination of bile acids with taurine (W. Wang et al., 2018). Elevated concentrations of taurocholic acid *in vivo* can elicit a certain degree of inflammatory response. Taurocholic acid levels were elevated in the NAFLD model in this study. However, a significant readjustment was observed after treatment.

Arachidonic acid regulates oxidative stress in the mitochondria of hepatocytes and exacerbates hepatocyte injury via three important metabolic pathways: lipoxygenase (LOX), cyclooxygenase (COX), and cytochrome plain (P450) (Trostchansky et al., 2021). An important metabolite of arachidonic acid is 12-HETE, which promotes neutrophil-induced inflammatory responses (Kriska et al., 2022). In this study, arachidonic acid content decreased in the model group, whereas the 12-HETE content increased relative to that in the control group, indicating dysfunctional arachidonic acid metabolism in NAFLD rats. After 12 weeks of treatment administration, there was a significant reversal in the levels of inflammation-related metabolites. Collectively, these results suggested that STM and RG improved the levels of metabolites associated with inflammation and reduced hepatic steatosis in patients with NAFLD. It was presumed, based on the results, that RG and STM have a facilitative effect on arachidonic acid metabolism-related enzymes and metabolite inhibitors. The findings of this study would provide theoretical guidance for treating NAFLD using herbal monomers.

Citric acid, fumaric acid, and ketoglutarate are key intermediates during the TCA cycle and also play important roles in glucose aerobic oxidation, amino acid metabolism, and lipid oxidation (Lin et al., 2020). Citrate synthase (CSY) present inside mitochondria is responsible for catalyzing the condensation of acetyl coenzyme A and oxaloacetate into citric CoA, which is then rapidly hydrolyzed to citric acid (S. Zhao et al., 2020). Isocitric acid is further converted to ketoglutarate under the action of isocitrate dehydrogenase (IDH). Fumaric acid is a precursor of L-malic acid, which is produced through the oxidation of succinic acid under the action of succinate dehydrogenase (SDH) (Balak et al., 2021). In this study, citric acid, ketoglutarate, and fumarate contents of the model group were apparently decreased relative to those of the control group, indicating metabolic dysregulation of the TCA cycle in HFD-induced NAFLD rats. This effect is associated with the inhibition of CSY, IDH, and SDH upon HFD feeding, which results in reduced synthesis of citric acid, ketoglutarate, and fumarate. The levels of citric acid, ketoglutarate, and fumaric acid significantly increased after STM and RG administration, which gradually increased hepatic mitochondrial production and reduced insulin resistance. Aspartic acid is synthesized from oxaloacetate. Aspartate levels in the NAFLD model group apparently increased, indicating that aspartate was being over-synthesized from oxaloacetate and that acetyl coenzyme A could not combine with sufficient oxaloacetate to enter the tricarboxylic acid cycle (J. Liu et al., 2021). Following treatment administration for 12 weeks, the aspartic acid level was markedly altered in the STM group and approached that in the control group.

As an essential amino acid, tryptophan can be metabolized by tryptophan-2,3-dioxygenase (TDO) and indoleamine-2,3-dioxygenase (IDO) into kynurenine and other metabolites. These two intracellular enzymes have multiple effects on the regulation of viral hepatitis, autoimmune liver disease, NAFLD, and cirrhosis (Zhou Q. et al., 2021). Tryptophan reduces inflammatory factors and liver fibrosis when it is metabolized to kynurenic acid. Moreover, 5-hydroxytryptamine is associated with oxidative stress, whereas tryptophan reportedly promotes 5-hydroxytryptamine production in the intestine (Choi et al., 2019). Abnormal tryptophan metabolic pathway leads to elevated lipid peroxidation levels in hepatocytes as well as increased damage to mitochondria (Kundi et al., 2021). This study revealed that the tryptophan metabolic pathway was disrupted in NAFLD model rats, resulting in a reduced tryptophan metabolic capacity and significantly upregulated levels of tryptophan. Tryptophan levels were progressively downregulated after STM and RG administration, which may be related to the promotion of IDO and TDO activities. Tryptophan level in the RG group was similar to that in the control group, indicating that RG could serve as a novel therapeutic approach against NAFLD.

D-Glucose serves as a primary energy source for the body, participating in glucose metabolism and insulin signaling pathways. In NAFLD, glucose metabolic dysregulation is prevalent, often manifesting as insulin resistance and increased hepatic gluconeogenesis. Insulin resistance impairs glucose utilization, leading to elevated blood glucose levels, while enhanced hepatic gluconeogenesis further exacerbates hyperglycemia. Concurrently, increased gluconeogenesis promotes fatty acid synthesis, intensifying hepatic lipid accumulation (Farías et al., 2024; Lee et al., 2025). Stearic acid and palmitic acid, common saturated fatty acids, are major components of dietary fats and also participate in endogenous fatty acid synthesis. In NAFLD, heightened hepatic fatty acid synthesis results in the accumulation of saturated fatty acids. Excessive saturated fatty acids can induce inflammatory responses and cell death in hepatocytes, exacerbating liver injury (Alves-Bezerra and Cohen, 2017).

Glycerophospholipids are important components of biological membranes and bile acids and are also involved in protein recognition and signaling in the cell membrane (Cui et al., 2020). Glycerophospholipids serve as precursors for diverse bioactive lipid signaling molecules, such as arachidonic acid, lysophosphatidic acid and platelet-activating factor. These lipid mediators participate in regulating a range of physiological and pathological processes, including inflammation and cell proliferation. Disturbances in glycerophospholipid metabolism may lead to disturbances in the energy metabolism of the body, causing liver detoxification dysfunction and fat accumulation, which is the main mechanism leading to NAFLD (Vvedenskaya et al., 2021). Lysophosphatidylcholine (LysoPC) is produced through hydrolysis in which an acyl chain is removed from phosphatidylcholine (PC), and this has a strong hemolytic effect and can cause necrosis of red blood cells. Compared to those in the control group, the PC level decreased, and the LysoPC level increased in the model group, indicating that the model rats exhibited disorders of glycerophospholipid metabolism. The levels of PC (36:3), LysoPC (15:0), PC (16:0/18:1(11Z)), and PC (18:0/20:3(5Z,8Z,11Z)) were reversed after the administration of STM and RG. The liver must package synthesized triglycerides into VLDLs and secrete them into the bloodstream to facilitate the export of fats from the liver. Deficiency of PC severely impairs VLDL secretion, leading to the accumulation of triglycerides within hepatocytes and driving the development of hepatic steatosis. The decrease in PC levels in the NAFLD model group led to a decrease in VLDL-C synthesis, resulting in the accumulation of TG in hepatocytes. This may be attributed to the extensive hydrolysis of PC in response to phospholipase A. Normal levels of glycerophospholipids promote lipoprotein production and transport *in vivo*. The experimental results in this study suggested that STM and RG improved the abnormal metabolism of glycerophospholipids, which then maintained the homeostasis of intracellular lipid metabolism in the rats.

The possible mechanism for the stereochemical differences of RS and SG on target binding was preliminarily discussed using molecular docking method (supporting information). Tryptophan metabolism directly influences intracellular NAD^+^ levels, and NAD^+^ serves as a critical coenzyme for the deacetylase activity of sirtuin 1 (SIRT1). Studies have demonstrated that SIRT1 maintains metabolic homeostasis by regulating mitochondrial function and oxidative stress responses. The hepatoprotective effects of taurine are associated with its capacity to ameliorate mitochondrial respiratory chain function and scavenge reactive oxygen species (ROS), a process that aligns closely with SIRT1-mediated antioxidant mechanisms, suggesting that taurine may exert its effects by modulating the SIRT1 signaling pathway. Additionally, the tricarboxylic acid (TCA) cycle dynamically regulates SIRT1 activity by altering the NAD^+^/NADH ratio. SIRT1 further inhibits sterol regulatory element-binding protein 1c (SREBP-1c) to reduce fatty acid synthesis and activates peroxisome proliferator-activated receptor α (PPARα) to promote fatty acid oxidation. Given the pivotal role of the tryptophan metabolism-NAD^+^-SIRT1 axis in energy metabolism and the modulatory properties of taurine on mitochondrial function, we selected SIRT1 as the molecular docking target. Molecular docking results of RG and SG with SIRT1 show that RG has better affinity than SG.

## 5 Conclusion

This study utilized non-targeted serum metabolomics to identify the biomarkers associated with STM, RG, and SG. In addition, the metabolic pathways associated with these biomarkers were revealed and analyzed. Blood glucose levels, lipid biochemical indices, and histopathology data were used as auxiliary tools to explore the pharmacological mechanisms of STM, RG, and SG in the treatment of NAFLD. A total of 21 differentially abundant metabolites associated with the effects were identified for the first time. Pathway enrichment analysis revealed that STM could treat NAFLD through the modulation of tryptophan metabolism, arachidonic acid metabolism, the TCA cycle, and glycerophospholipid metabolism. RG could exert therapeutic effects on NAFLD through the regulation of taurine and hypotaurine metabolism, the TCA cycle, arachidonic acid metabolism, tryptophan metabolism, and glycerophospholipid metabolism. These findings revealed the mechanism through which STM and RG regulate NAFLD, suggesting these two as potentially effective drugs for NAFLD improvement. The administration dose of RG will be optimized in our future research for possible clinical application. Meanwhile, the stereochemical differences of RG and SG on target binding such as differences in enzyme affinity will be studied to deepen the mechanistic research in the future.

## Data Availability

The original contributions presented in the study are publicly available. This data can be found here: 10.6084/m9.figshare.30110707.

## Glossary

HFD: high-fat diet
STM: swertiamarin
RG: *(R)*-gentiandiol
SG: *(S)*-gentiandiol
NAFLD: Non-alcoholic fatty liver disease
NASH: non-alcoholic steatohepatitis
HCC: hepatocellular carcinoma
**ESI** ^**+**^: positive ionisation mode and ESI^−^ negative ionisation mode
UPLC-Q-TOF/MS: Ultra Performance Liquid Chromatography-Quadrupole-Time Of Flight-Mass Spectrometry
KEGG: Kyoto encyclopaedia of Genes and Genomes
TCM: Traditional Chinese Medicine
LC-MS: Liquid Chromatograph Mass Spectrometer
TC: total cholesterol
TG: triglyceride
ALT: alanine aminotransferase
AST: aspartate aminotransferase
LDL-C: low-density lipoprotein cholesterol
HDL-C: high-density lipoprotein cholesterol
MDA: malondialdehyde
SOD: superoxide dismutase
GSH-PX: glutathione peroxidase
HE: hematoxylin-eosin
AD: asymmetric dihydroxylation
UPLC: ultra-performance liquid chromatography
QC: Quality Control
PCA: principal component analysis
PLS-DA: partial least squares discriminant analysis
OPLS-DA: orthogonal partial least squares discriminant analysis
VIP: variable importance in projection value
ANOVA: analyzed by one-way analysis of variance
CDO: Cysteine dioxygenase
